# Communal coping and its association with marital relations and psychological outcomes among healthcare professionals during the COVID-19 pandemic

**DOI:** 10.3389/fpsyg.2022.936108

**Published:** 2022-07-22

**Authors:** Zainab Alimoradi, Mohammad Ali Soleimani, Maryam Keramtkar, Nasim Bahrami, Mark D. Griffiths

**Affiliations:** ^1^Social Determinants of Health Research Center, Research Institute for Prevention of Non-communicable Diseases, Qazvin University of Medical Sciences, Qazvin, Iran; ^2^Department of Psychology, Nottingham Trent University, Nottingham, United Kingdom

**Keywords:** healthcare professionals, communal coping, COVID-19 pandemic, psychological distress, dyadic adjustment

## Abstract

**Background:**

Communal coping is a type of interdependency in which couples dealing with a health threat share assessment of a threat and respond together to the stress. The present study investigated communal coping in the COVID-19 pandemic and its association with psychological and relational outcomes among healthcare professionals.

**Methods:**

In the present cross-sectional survey study, 242 healthcare professionals from hospitals and health centers were recruited *via* convenience sampling between August and October 2020. Communal coping with working conditions during the COVID-19 pandemic, dyadic adjustment, psychological distress, and fear of COVID-19 along with demographic and professional characteristics were assessed via an online survey.

**Results:**

Multivariable linear regression showed that dyadic adjustment (β = 0.73), psychological distress (β = 0.16), fear of COVID-19 (β = 0.11), and support gap (β = −0.04) were significant independent variables associated with communal coping among healthcare professionals.

**Conclusion:**

Healthcare professionals coped communally within the family in dealing with working conditions during the COVID-19 pandemic. Dyadic adjustment was the strongest predictor of communal coping among healthcare professionals.

## Introduction

The world has faced the COVID-19 pandemic since early March 2020. The sharp rise in morbidity and mortality of COVID-19 resulted in high stress in the community as well as healthcare professionals who needed to cope with the healthcare of patients with COVID-19 (Wu et al., 2020). Coping has been predominantly examined from an individualistic approach (Afifi et al., 2006). Now, research on coping has shifted from considering coping as a primarily individual phenomenon toward a more interdependent process. According to systems theory, it is difficult to isolate and analyze individual coping from the coping of family members because coping takes place in an interpersonal context (Lyons et al., 1998). In addition, most life stressors are interpersonal, and coping requires interaction with others (Monnier and Hobfoll, 1997).

Relationship-focused coping refers to modes of coping with the aim of managing, preserving, and maintaining relationships during stressful periods. Past research supports the effectiveness of these strategies, particularly in the context of communal stressors (O’Brien and DeLongis, 1997). Individuals manage stress in the context of interpersonal relationships including family relationships (Lyons et al., 1998; Afifi et al., 2006). In addition, individual coping has a broader social impact including how it affects their families and partners (Monnier and Hobfoll, 1997). Studies have also shown that couples act as an interpersonal system in coping. Marriage is a dyadic relationship and couples have a mutual effect on each other’s behavior (Durtschi et al., 2011). From this perspective, the social context of coping should be taken into consideration as no one is completely self-sufficient. Partners in a dyad must be considered as an interdependent whole in which each partner influences the other (Bodenmann et al., 2006).

When social groups experience shared stressors, communal coping can be an effective form of coping (Lyons et al., 1998). Communal coping is one of the interpersonal theoretical perspectives on coping developed by Lyons et al. (1998). Communal coping is considered one form of relationship-focused coping. Relationship-focused coping types are categorized by participants’ appraisal of the stressor and the action taken in response to the stress. Communal coping is a type of interdependency in which couples dealing with a health threat share assessment of a threat and respond together to the stress (Lyons et al., 1998). Therefore, communal coping occurs along two dimensions of appraisal and action (Lyons et al., 1998; Afifi et al., 2006). For communal coping, individuals appraise stressors as a shared problem rather than the one to be dealt with alone and collaborate in managing the stressor (Lyons et al., 1998). Appraisal, which addresses ownership of the stressor, involves individuals’ perception that the stressor is “our problem” to deal with rather than “your problem.” Action addresses responsibility of the stressor in which individuals considered the stressor as “our responsibility” (Lyons et al., 1998; Afifi et al., 2006). Therefore, communal coping involves shared appraisal and joint action rather than individual appraisal (Lyons et al., 1998) to manage the stressor in the context of both one’s own and others’ needs (Lawrence and Schiller Schigelone, 2002).

Individuals engage in communal coping because it has some benefits that are not gained by acting alone. Communal coping expands resources and capacity for coping with stress, social support, and quality of relationships (Lyons et al., 1998). Communal coping occurs when many individuals face the same stressor and engage in joint action to manage it. Stressful events that simultaneously affect the whole community may naturally induce the community to cope together (Lyons et al., 1998). Fino et al. (2021) reported that psychological distress was lower among nurses who tried to help patients with COVID-19 communicate with their families. Consequently, the COVID-19 pandemic may have affected the use of communal coping. Although some studies have examined communal coping with health-related issues in long-term conditions, no study has examined this coping pattern during a pandemic.

### Aim of the present study

The present study examined how healthcare professionals consider the COVID-19 pandemic as a shared problem within their family relationships (and more specifically their spouse) and how they dealt with it. Given a variety of benefits of employing communal coping, the study investigated how this pattern was related to marital relations and psychological outcomes.

## Methods

### Design

The present study was a cross-sectional survey conducted from August to October 2020.

### Participants

**FIGURE 1:**
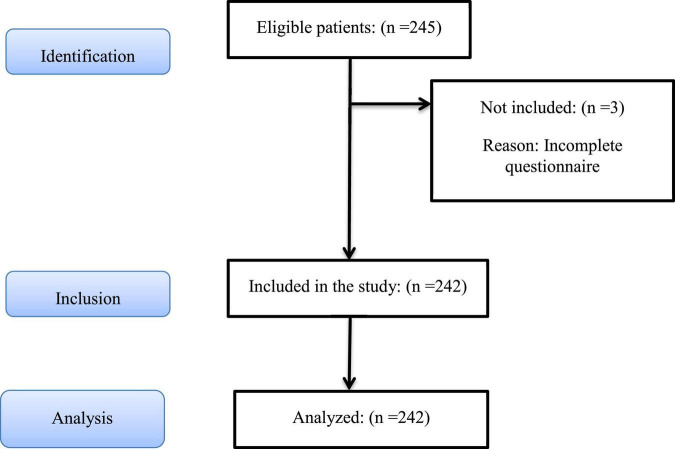
Study recruitment procedure based on STROBE flow diagram.

The participants comprised 245 healthcare professionals working at hospitals and health centers. Participants were recruited from six public hospital and three private hospitals and 12 comprehensive health centers. During the COVID-19 pandemic, one of these public hospitals was assigned as the referral center for COVID-19 patients. Two other hospitals also provided healthcare to patients with COVID-19 at the peak time. Figure 1 describes the recruitment procedure. All married staff working at hospitals and health centers with at least 6 months of working experience were eligible to participate in the study. Those who were single, divorced, or living away from their spouse were excluded from the study as the present study examines coping at the dyadic level.

### Sampling and recruitment

Participants were selected by convenience sampling. The link of the study was sent to potential participants in which inclusion criteria and aim of the study were explained. After agreeing to participate in the study, the link to the online survey was sent to them. Sample size was calculated with two approaches. The minimum sample size for regression model should be 10 individuals per variable (Green, 1991). As 20 variables were entered into the model, at least 200 participants were required for an adequate sample size. In the second approach, considering α = 0.05, power of 80%, and minimum correlation of 0.2 between variables, sample size was estimated to be 195 individuals.

### Variables and measures

Data were collected using an online survey hosted on the *Porsline* platform. The link to the online survey was sent to potential participants *via* social media apps (*WhatsApp* and *Telegram*), SMS, and emails to the participants. Data were collected using the following measures and psychometric scales:

#### Socio-demographic characteristics

The following socio-demographic data were collected: age, educational level, spouse’s age and educational level, working condition, work experience, and marital relationship duration.

#### Communal coping in the pandemic

This variable was assessed using the seven-item Communal Coping Scale (CCS; adjusted for working during the COVID-19 pandemic). The items (e.g., *“My spouse and I talk about how to deal with this situation”*) were adapted from Afifi et al. (2006). Participants were asked to think about the extent to which they and their partner coped communally with the COVID-19 pandemic on a five-point scale from 1 (*strongly disagree*) to 5 (*strongly agree*). The final score was calculated based on the total scores of the items and ranged from 7 to 35. Higher scores indicate a higher level of communal coping.

The scale was adapted to the study conditions (i.e., working during the COVID-19 pandemic in the healthcare system). Its validity was evaluated by qualitative face validity and content validity by 15 faculty members of the School of Nursing and Midwifery. Then, the construct validity was investigated using exploratory factor analysis (EFA). As the Kaiser-Meyer-Olkin (KMO) index of 0.75 with a significant correlation between pairs of variables based on Bartlett sphericity test (*p* < 0.001) was acquired, EFA was performed (Kellar and Kelvin, 2013). EFA using varimax rotation and scree plot verified that the scale had two factors. These factors were named “Shared appraisal” and “Collaborative action,” the same as the previous study of communal coping among couples with health problems (Rentscher, 2019). Based on EFA, the Communal Coping Scale (adjusted for working during the COVID-19 pandemic) with two factors explained 78.82% of variance. In the present study, the internal reliability of the scale was very good (Cronbach’s alpha = 0.89).

#### Dyadic adjustment

This variable was assessed using the 14-item Dyadic Adjustment Scale comprising three subscales: dyadic satisfaction, dyadic cohesion, and dyadic consensus. The items (e.g., *“Have you ever regretted getting married?”*) are rated on a six-point scale (range from 0 to 5). The total scores range from 0 to 84 with a higher score indicating better marital adjustment. This scale has shown good content validity, criterion-related validity, and construct validity with high scale reliability (Busby et al., 1995). Good psychometric properties of the Persian version have been reported (Isanezhad et al., 2012). In the present study, the internal reliability of the scale was excellent (Cronbach’s alpha = 0.91).

#### Support gap

This variable assessed five items adapted from the Emotional and Esteem subscales of Xu and Burleson’s Spousal Support Measure (Xu and Burleson, 2001). Participants are asked to reflect on conversations with their partner and to estimate how often they received different types of reactions from him or her (e.g., *“Tells you that he loves you and is close to you”*). The items are rated on a five-point scale from 1 (*never*) to 5 (*all the time*). Participants are asked to rate how much they desired different reactions from their spouse, using the same scales. Support gap was calculated as the difference between participants’ desired and received support. Support gaps included positive scores (desiring more than one received) and negative scores (receiving more than desired) (Xu and Burleson, 2001). Smith et al. (2018) reported the scale had good reliability. The scale was translated into Persian, and its qualitative face validity and content validity were verified based on comments from 15 faculty members of the School of Nursing and Midwifery. The construct validity was then investigated using EFA. As the KMO index of 0.92 with a significant correlation between pairs of variables based on Bartlett sphericity test (*p* < 0.001) was acquired, EFA was performed (Kellar and Kelvin, 2013). EFA using varimax rotation and scree plot verified that this scale had two factors of “perceived support” and “expected support.” EFA confirmed that this scale explained 84% variance in support gap. In the present study, the internal reliability of the scale was excellent (Cronbach’s alpha = 0.94).

#### Psychological distress

This variable was assessed using the 10-item K-10 Psychological Distress Questionnaire (Drapeau et al., 2012). The items (e.g., *“During the past month, how often did you feel nervous?”*) are rated on a five-point scale from 1 (*never*) to 5 (*always or all the time*). The scores range from 10 to 50. Higher scores indicate greater levels of psychological distress (Kessler et al., 2003). Good psychometric properties of the Persian version have been reported (Yaghubi, 2016). In the present study, the internal reliability of the scale was excellent (Cronbach’s alpha = 0.94).

#### Fear of COVID-19

This variable was assessed using the seven-item Fear of COVID-19 Scale (Ahorsu et al., 2020). The items (e.g., *“It make me uncomfortable to think about COVID-19”*) are rated on a five-point scale from 1 (*strongly disagree*) to 5 (*strongly agree*). The total scores range from 7 to 35. A higher score indicates greater fear of COVID-19. Good psychometric properties of the Persian version have been reported (Ahorsu et al., 2020). In the present study, the internal reliability of the scale was very good (Cronbach’s alpha = 0.88).

### Ethical considerations

The study protocol was approved by the institutional research review board and regional Ethics Committee of Biomedical Research affiliated with Qazvin University of Medical Sciences (reference code: IR.QUMS.REC.1399.174). After explaining the purpose of the study, and assuring the privacy and confidentiality of the data, written informed consent was obtained from all participants.

### Data analysis

Data were analyzed using SPSS software version 24. Categorical variables were described with frequencies and percentages and continuous quantitative variables were described using means and standard deviations (SDs). Univariable and multivariable linear regression models were used to investigate the association between communal coping and psychological variables (e.g., psychological distress and fear of COVID-19), and spouse’s relational variables (e.g., dyadic adjustment and support gap). In all regression models, the total score of communal coping was entered as a dependent variable and other variables were entered as independent variables. Independent variables that had a significant level of less than 0.05 in the univariable linear regression model were included in the multivariable model *via* a stepwise approach. Considerations of using linear regression method including normal distribution of dependent variable, outlier data, and collinearity between independent variables (based on VIF < 10) were controlled for. The significance level was considered to be *p* < 0.05.

## Results

The mean age of participants in the present study was 37.40 years (SD = 7.80). The majority of participants were women (79.8%) and the mean age of their spouses was 39.14 years (SD = 8.41). The mean number of years’ work experience was 12.80 years (SD = 7.36). Almost half of the participants had a bachelor’s degree (52.9%). Among the participants, 28.5% were working in the inpatient COVID-19 wards and 8.7% in outpatient wards in which they visited suspected COVID-19 patients or followed up those during treatment. Examining the relationship between communal coping with demographic characteristics showed that variables of having responsibility for caring for a patient with COVID-19, spouse’s employment in health wards, and spouse’s health status had a significant relationship with communal coping. Demographic characteristics are presented in Table 1.

**TABLE 1:** Socio-demographic and main independent variables and univariable logistic regression analysis considering communal coping as a dependent variable.

Qualitative variables		No (%)	Univariable linear regression analysis		
			B	Std. error	*p*
Gender	Male	49 (20.2)	RG		
	Female	193 (79.8)	−0.58	0.9	0.52
Level of education	Technician	14 (5.8)	RG		
	B.Sc.	128 (52.9)	−2.06	1.57	0.19
	M.Sc.	60 (24.8)	−1.14	1.66	0.50
	Ph.D.	22 (9.1)	−0.97	1.91	0.61
	General/Specialist practitioner	18 (7.4)	−1.01	1.99	0.61
Spouses’ level of education	Technician	51 (21.1)	RG		
	B.Sc.	110 (45.5)	0.61	0.95	0.21
	M.Sc.	45 (18.6)	0.76	1.14	0.51
	Ph.D.	18 (7.4)	2.11	1.53	0.17
	General/Specialist practitioner	18 (7.4)	2.62	1.53	0.09
Working health sector	Comprehensive health clinic	60 (24.8)	RG		
	COVID-19 ward-Hospital	69 (28.5)	0.001	0.99	0.99
	General ward-Hospital	92 (38)	−0.24	0.93	0.80
	Outpatient COVID-19 Clinic	21 (8.7)	−1.05	1.42	0.46
Spouses’ job	Unemployed	27 (11.2)	RG		
	Employed	204 (84.3)	0.03	1.14	0.98
	Retired	11 (4.5)	2.53	1.10	0.21
Spouse working in health sectors	No	177 (73.1)	RG		
	Yes	65 (26.9)	1.42	0.80	0.11
Spouses’ working health sector	Not applicable	185 (76.4)	RG		
	Comprehensive health clinic	14 (5.8)	1.30	1.54	0.40
	COVID-19 ward–hospital	18 (7.4)	2.84	1.37	0.04
	General ward–hospital	19 (7.9)	1.39	1.34	0.30
	Outpatient COVID-19 clinic	6 (2.5)	3.57	2.30	0.12
Health status	Weak	9 (3.7)	RG		
	Fair	73 (30.2)	−2.75	1.96	0.16
	Good	160 (66.1)	−1.22	1.90	0.52
Spouses’ health status	Weak	7 (2.9)	RG		
	Fair	72 (29.8)	−4.06	2.17	0.06
	Good	163 (67.4)	−1.65	2.12	0.44
Exposure to patient with COVID-19	No	142 (58.7)	RG		
	Yes	100 (41.3)	1.50	0.72	0.04
History of COVID-19	Not infected	168 (69.4)	RG		
	Infected and recovered	53 (21.9)	0.97	0.88	0.27
	Infected and under treatment	5 (2.1)	−3.95	2.53	0.12
	Suspected	16 (6.6)	−0.52	1.46	0.72
Quantitative variables	Range	Mean (SD)	B	Std. Error	*p*
Age (year)	23–67	37.4 (7.80)	0.009	0.05	0.84
Spouse age (year)	24–68	39.14 (8.41)	−0.004	0.04	0.93
Marital duration (year)	1–41	11.43 (7.94)	−0.04	0.05	0.33
Working experience (in years)	1–37	12.80 (7.36)	−0.006	0.05	0.90
Fear of COVID-19	7–35	18.43 (6.61)	1.34	0.04	<0.001
Psychological distress	10–50	23.50 (8.75)	1.04	0.03	<0.001
Dyadic adjustment	0–69	45.17 (12.56)	0.60	0.009	<0.001
Support gap (received–expected)		−2.13 (4.94)	−1.92	0.32	<0.001
Received support	5–25	18.45 (5.62)			
Expected support	5–25	20.57 (5.32)			
Communal coping	7–35	28.25 (5.58)			

RG, reference group.

The mean communal coping score on the CCS was 28.25 (SD = 5.58). Given the scores range from 7 to 35, the average score of the participants was more than 75% of the total score, it appears that communal coping was used a lot by the participants. The mean scale score was 18.43 out of 35 (SD = 6.61) for fear of COVID-19, 23.50 out of 50 (SD = 8.75) for psychological distress, 18.45 out of 25 (SD = 5.62) for received support, 20.57 out of 25 (SD = 5.32) for expected support, and 45.17 out of 84 (SD = 12.56) for dyadic adjustment. All of these variables had a significant relationship with the communal coping in the univariable linear regression model and were selected to enter the multivariable regression model (Table 1).

Results of multivariable regression showed that the independent variables of dyadic adjustment, psychological distress, fear of COVID-19, and support gap were significantly associated with communal coping. According to standardized beta coefficients in the multivariable model, dyadic adjustment (β = 0.73) was the strongest independent predictor of communal coping among healthcare professionals (with a direct moderate to large association). Psychological distress with a standardized beta coefficient of 0.16 (with a direct weak association), fear of COVID-19 with a coefficient of 0.11 (with a direct weak association) and support gap with a coefficient of −0.04 (with a very weak reverse association) also predicted communal coping. However, they had less power in predicting participants’ communal coping. In total, the variables in this model explained 96% of variance of communal coping (Table 2).

**TABLE 2:** Results of multivariable logistic regression analysis considering communal coping as a dependent variable.

	Unstandardized coefficients		Standardized coefficients	Sig.	95.0% confidence interval for B	
	B	Std. error	Beta		Lower bound	Upper bound
Dyadic adjustment	0.45	0.02	0.73	<0.001	0.41	0.48
Psychological distress	0.18	0.04	0.16	<0.001	0.10	0.26
Fear of COVID-19	0.16	0.06	0.11	0.006	0.05	0.27
Support gap	−0.20	0.07	−0.04	0.009	−0.34	−0.05
Model summary	R: 0.98; R^2^: 0.96; Adjusted R^2^: 0.96; Durbin-Watson: 2.08					

## Discussion

The present study examined communal coping among healthcare professionals and its relationship with psychological distress and relational characteristics during the COVID-19 pandemic. Results of the present study showed that participants have used communal coping in the COVID-19 pandemic situation. Dyadic adjustment, psychological distress, support gap, and fear of COVID-19 were independent variables that significantly predicted communal coping.

Communal coping has primarily been examined at the community level in natural disasters (Wlodarczyk et al., 2016) and at communal settings such as aging in retirement communities (Lawrence and Schiller Schigelone, 2002) and living in refugee camps (Afifi et al., 2019). It has also been investigated at the relational context of family experiencing life events such as pregnancy (Monnier and Hobfoll, 1997) and divorce (Afifi et al., 2006). These studies have focused on coping with collective stressors in which many individuals face the same stressor and engage in joint action to manage it. Evidence suggests that communal coping is beneficial to individuals dealing with stressors. Studies have shown that communal coping enhances mental health among pregnant women (Monnier and Hobfoll, 1997) and adolescents dealing with uncertainty of living in refugee camps (Afifi et al., 2019); psychological adjustment to the genetic risk of cancer in family members (Koehly et al., 2008); and recovery (Afifi et al., 2012) and posttraumatic growth from natural disasters (Wlodarczyk et al., 2016). These studies have focused on the impact of communal coping on their own or others’ adjustment, which are consistent with the findings of the present study. Although the majority of these studies have been conducted in both short-lived and long-term stressful situations, less research has been conducted on health issues including chronic conditions.

Dyadic adjustment was the strongest independent variable in relation to communal coping among healthcare professionals. Couple adjustment is a continuous and changing process (Manyam and Junior, 2014). Marital adjustment is a situation in which couples often feel happy and satisfied with each other and is formed through mutual interest, mutual care, acceptance, understanding, and satisfaction of each other’s needs (NasrollahiMola et al., 2020). Having a sense of mutual care as one of the dimensions of dyadic adjustment helps explain the significant relationship between marital adjustment and communal coping among couples working in high-risk conditions during the COVID-19 pandemic.

Spouses’ dyadic adjustment is an active process in which couples gradually find their role in the family and understand their responsibilities (Umberson et al., 2005). Since the mean marital duration was approximately 11 years among participants, it seems that they had enough time to achieve marital adjustment in cohabitation. The relationship between marital adjustment and duration of marriage was investigated in an exploratory manner using univariable regression in the present study. The results showed that each year of increase in marital duration, a 2.7 increase was observed in dyadic adjustment. Also, the average score of the participants on the Dyadic Adjustment Scale (45.17) was close to two-thirds of the maximum score (i.e., 46 out of 69). Therefore, it seems that the healthcare professionals in the present study did not consider working during the COVID-19 pandemic to be an individual problem. They talked about it together and tried to develop solutions to work-related problems with consequences such as psychological distress.

Psychological distress and fear of COVID-19 also predicted communal coping. Given the positive association between fear of COVID-19 and psychological distress and communal coping, it seems that with increasing fear of COVID-19 and psychological distress, communal coping increased. Communal coping may have increased as a compensatory mechanism in response to psychological distress. However, due to the cross-sectional nature of the study, it is not possible to determine the precedence or latency of the relationship between these variables. In communal coping, individuals deal with the shared appraisal of the current situation and then try to cope with the situation with collaborative action. Working during the COVID-19 pandemic among healthcare professionals can lead to increased psychological distress due to increased exposure to COVID-19 and an increased risk of personal injury.

The sudden onset of the COVID-19 pandemic put considerable pressure on healthcare professionals (Liu et al., 2012). Studies have also shown an increase in psychological distress, depression, anxiety, and stress among healthcare professionals during the COVID-19 pandemic (Levin, 2019; Wang et al., 2020). In addition to high stress associated with constant exposure to COVID-19 patients, healthcare professionals have also been concerned about maintaining their own and their families’ health (Medic et al., 2017). Despite extensive research, no previous study has examined the status of marital variables among healthcare professionals during the COVID-19 pandemic. In previous studies, an inverse relationship was reported between communal coping and psychological distress (Berg et al., 2008; Karan et al., 2019), which was inconsistent with the results of the present study. The main difference between the present study and these studies may be that in previous studies, participants’ psychological distress was due to one of the types of chronic diseases that have no cure and the individual may be affected for the rest of their life. However, in the present study, psychological distress was likely caused by a person’s work conditions that were not stable. According to the results of the present study, it appears that when individuals have higher dyadic adjustment, in the face of critical situations such as the COVID-19 pandemic, which in particular increases the risk of disease for healthcare professionals and their families, receive more support from their spouses and show better coping.

The present study showed a negative and significant relationship between support gap by the spouse and communal coping. This means that with increasing support gap from the spouse, communal coping decreased significantly. Existence of a significant inverse relationship between spouses’ support gap and communal coping in line with other findings of present study point to the importance of intimate relationships between spouses to achieve a better situation in communal coping during a crisis.

### Strengths and limitations

To the best of the present authors’ knowledge, the study here is one of the first to examine communal coping of healthcare workers working during the COVID-19 pandemic. Some of strengths of the current study are appropriate sample size, variation in participants based on the working status as healthcare professionals, and application of both univariable and multi-variable statistical analyses. In interpreting the findings of the present study, its limitations should be considered. Using self-report measures and lack of dyadic data collection are among the limitations of the present study. Having a cross-sectional design means the precedence and latency of the relationship between the variables are unclear. Due to the pandemic, the aim of the present study was to evaluate the status of communal coping among healthcare professionals, but due to the cross-sectional nature of the study, the causal relationship between the variables cannot be assessed. Another limitation was that in the present study, dyadic adjustment was examined only among married couples and relationships with a partner other than the spouse could not be examined due to the cultural conditions of the Iranian community.

## Conclusion

Results of the present study indicated that healthcare professionals coped communally in the family in dealing with working during the COVID-19 pandemic. Dyadic adjustment was the strongest independent variable that predicted communal coping among healthcare professionals working during the pandemic of COVID-19. Given that marital adjustment was the strongest predictor of communal coping, it seems that preventive interventions to promote marital relationships by family consultants or psychologists can act as a positive reinforcer to promote communal coping. The stress of healthcare professionals was exacerbated by special circumstances such as the COVID-19 pandemic. Even in non-pandemic situations, the nature of work related to providing health services is stressful, and strengthening couples’ relationships as a buffer will help individuals in the face of adversity. The present study showed that social domain of coping should be taken into consideration in the context of collective stressors.

## Data availability statement

The raw data supporting the conclusions of this study will be made available by the authors, without undue reservation.

## Ethics statement

The studies involving human participants were reviewed and approved by the Ethical committee affiliated with Qazvin University of Medical Sciences. The participants provided their written informed consent to participate in this study.

## Author contributions

NB, MS, and ZA contributed to data gathering and preparing data for analysis. ZA and MS contributed to data analysis and interpretation of findings. NB and ZA drafted the original manuscript. MS and MK provided contributions to the literature review, substantially edited the primary manuscript, and prepared the final version of the manuscript. MDG redrafted the revised version and thoroughly revised and edited the final version of the manuscript at the revision stage. All authors contributed to the conception and design of the study except MDG. All authors revised the manuscript, agreed to be fully accountable for ensuring the integrity and accuracy of the study, and read and approved the final version of the manuscript to be published.

## Conflict of interest

The authors declare that the research was conducted in the absence of any commercial or financial relationships that could be construed as a potential conflict of interest.

## Publisher’s note

All claims expressed in this article are solely those of the authors and do not necessarily represent those of their affiliated organizations, or those of the publisher, the editors and the reviewers. Any product that may be evaluated in this article, or claim that may be made by its manufacturer, is not guaranteed or endorsed by the publisher.
